# Evaluating the Efficacy and Safety of Radiofrequency Ablation Compared to Endoscopic Surveillance in Managing Low-Grade Dysplasia in Barrett’s Esophagus: A Systematic Review and Meta-Analysis

**DOI:** 10.7759/cureus.96304

**Published:** 2025-11-07

**Authors:** Yara K Alzahrani, Areej A Abu Deeb, Banan A Abdulghafur, Mohammed H Kattouah, Taif M Al-Waghdani, Doaa Alqaidy

**Affiliations:** 1 College of Medicine and Surgery, King Abdulaziz University Faculty of Medicine, Jeddah, SAU; 2 General Surgery, King Fahad University Hospital, Al-Khobar, SAU; 3 College of Medicine and Surgery, Umm Al-Qura University, Makkah, SAU; 4 Pathology Department, King Abdulaziz University, Jeddah, SAU

**Keywords:** complete eradication of dysplasia (ce-d), complete eradication of intestinal metaplasia (ce-im), endoscopic surveillance (es), esophageal adenocarcinoma (eac), high-grade dysplasia (hgd), low-grade dysplasia in barrett’s esophagus (be-lgd), radiofrequency ablation (rfa)

## Abstract

Barrett’s esophagus with low-grade dysplasia (BE-LGD) risks progression to high-grade dysplasia (HGD) or esophageal adenocarcinoma (EAC). Guidelines remain equivocal on managing non-nodular BE-LGD, endorsing either radiofrequency ablation (RFA) or endoscopic surveillance (ES). This systematic review compares RFA and ES in preventing progression to HGD/EAC to inform clinical practice.Following the Preferred Reporting Items for Systematic Reviews and Meta-Analyses (PRISMA) guidelines, MEDLINE, EMBASE, Web of Science, and Google Scholar were searched (September 2024) for randomized controlled trials (RCTs) comparing RFA and ES in BE-LGD. Outcomes included HGD/EAC progression, complete eradication of dysplasia (CE-D) and intestinal metaplasia (CE-IM), adverse events, and predictors of progression/response. Risk of bias (RoB) was assessed using Cochrane RoB 2. Meta-analysis used Mantel-Haenszel random-effects models.Four RCTs (n = 282) were included. The studies varied in follow-up duration and excluded patients with nodular BE-LGD. RFA was associated with reduced progression to HGD/EAC compared with ES (relative risk (RR) 0.23; 95% confidence interval (CI) 0.05-1.02; p = 0.05) and achieved higher CE-D and CE-IM both post-treatment (CE-D: RR 2.70; 95% CI 1.37-5.32; p = 0.004; CE-IM: RR 21.80; 95% CI 4.56-104.29; p < 0.0001) and durably (CE-D: RR 3.06; 95% CI 2.07-4.52; p < 0.00001; CE-IM: RR 61.60; 95% CI 8.66-438.21; p < 0.0001). Adverse events occurred only with RFA but were manageable. Single-study data reported that ES progression correlated with shorter Barrett’s diagnosis time, more dysplasia-positive endoscopies, and longer circumferential Barrett’s length. No significant predictors of RFA response emerged.In conclusion, RFA significantly reduces HGD/EAC progression in BE-LGD compared to ES, with superior CE-D/CE-IM rates and manageable adverse events, supporting its use as first-line therapy for non-nodular BE-LGD. Future studies should compare RFA to emerging therapies. The trials’ restrictive eligibility criteria, variable center and operator expertise, differences in treatment protocols and ablation targets, and methodological issues-including early exclusions, cross-over, incomplete reporting, and potential conflicts of interest-limited generalizability and interpretation of long-term efficacy and safety.

## Introduction and background

In Barrett’s esophagus (BE), the usual squamous epithelium lining the distal esophagus is replaced by columnar epithelium in response to chronic gastroesophageal reflux, a histological change called intestinal metaplasia (IM) [1]. This represents a key step in the metaplasia-dysplasia-carcinoma sequence that underlies the development of esophageal adenocarcinoma (EAC). Over time, neoplastic progression advances nondysplastic IM through stages of low-grade dysplasia (LGD) and high-grade dysplasia (HGD), eventually leading to EAC [2]. However, the histologic distinction between reactive or inflammatory changes and true LGD can be subtle, leading to interobserver variability in diagnosis and influencing subsequent management decisions [3].

In line with the American Gastroenterological Association (AGA) clinical practice guideline, the management of BE-LGD without a visible lesion remains conditional. Consequently, treatment should reflect patient preference- to minimize procedural risks via surveillance or to obtain potential benefits through endoscopic eradication therapy [4]. This poses the question: How efficacious and safe is radiofrequency ablation (RFA) compared to endoscopic surveillance (ES) in managing BE-LGD?

Given the potential for disease progression and the diagnostic uncertainty that can accompany LGD, determining the most effective management approach is essential to balance the risks of overtreatment and undertreatment. While several reviews have compared RFA and surveillance, few have synthesised long-term follow-up data assessing the durability of complete eradication (CE) outcomes. Accordingly, this systematic review will examine the efficacy of RFA compared to ES in preventing progression of BE-LGD to HGD or EAC. Secondary outcomes include rates of dysplasia and metaplasia eradication, treatment safety, and predictors of disease progression or therapeutic success.

## Review

Methods and materials 

Study Searching and Search Strategy

In this review, we followed the Preferred Reporting Items of Systematic Reviews and Meta-Analyses (PRISMA) model [5] to ensure that studies were selected with minimal bias. This study’s protocol was registered at PROSPERO [6] with the following ID: CRD42024584158. Protocol amendments are documented in the PROSPERO registry. Ethical approval was not required for this systematic review. In September 2024, we conducted a systematic search of the following databases: (1) MEDLINE (PubMed), (2) EMBASE, (3) Web of Science Core Collection, and (4) Google Scholar. All searches were conducted using the following Boolean string (applied identically across databases): (Barrett’s Esophagus OR BE OR Barrett Esophagus) AND (Radiofrequency Ablation OR RFA OR Ablation) AND (Endoscopic Surveillance OR Endoscopy OR Monitoring) AND (Low-Grade Dysplasia OR LGD). Studies were considered for the review based on the PICO (population, intervention, comparison, outcome) criteria. Specifically, studies considered included patients with a confirmed diagnosis of BE-LGD treated with RFA and compared with ES without RFA, assessing the rate of progression to HGD or EAC and the rate of CE-D or CE-IM. 

Methodology for Selecting Studies

Studies published without a time frame limitation were included in this review. The following criteria dictated the inclusion of studies: (1) Only RCTs were included to maximize internal validity and causal inference. We acknowledge this reduces generalizability and excludes large observational registry datasets. We also included (2) studies in English, Arabic, or German, (3) studies that reported the number of patients who underwent RFA and others who were monitored endoscopically only, and (4) studies that reported outcomes of interest relevant to clinical questions, such as (a) rate of progression from LGD to HGD or EAC, (b) rate of CE-D or CE-IM, and (c) relevant adverse events. By contrast, studies were excluded from our review based on the following criteria: (1) non-RCTs (such as cohorts and animal studies); (2) studies not in English, Arabic, or German; (3) studies involving fewer than ten patients; (4) studies with patients receiving combined treatment other than RFA or surveillance; (5) studies with patients first diagnosed with HGD or EAC; (6) studies where patients did not have RFA or ES; (7) studies that did not report the outcome of interest for the clinical question; and (8) duplicates of studies after screening for duplication. 

Process of Screening and Data Extraction

Three reviewers (BA, MK, and TA) screened papers simultaneously and independently by title and abstract using the Rayyan software [7]; differences were resolved by a fourth reviewer (YA). Then, the full texts were reviewed by three independent reviewers (YA, BA, and MK) simultaneously, and differences were resolved by a fourth reviewer (AA). Afterward, two reviewers (AA and TA) performed data extraction for the following variables: (1) study setting, (2) total sample size, (3) sample size receiving RFA, (4) sample size under ES, (5) follow-up duration, (6) number of RFA sessions for each (RFA and ES sample size); (a) under demographic and disease-specific characteristics of enrolled patients: (7) age, (8) number of male patients, (9) BMI, (10) ethnicity, (11) BE length (maximum and circumferential lengths), (12) time since diagnosis of BE, (13) time since diagnosis of dysplasia; (b) under primary and secondary efficacy outcomes: (14) number of patients who progressed from LGD to HGD, (15) number of patients who progressed from LGD to EAC, (16) post-treatment CE-D, (17) post-treatment CE-IM, (18) durable CE-D, (19) durable CE-IM, and (20) adverse events. Further variables obtained include (21) study predictors of progression under ES, (22) study predictors of response to RFA, (23) study strengths, (24) study limitations, and (25) eligibility criteria. Finally, the retrieved data were double-checked.

Assessment of Quality and Bias Risk

The risk of bias (RoB) was independently assessed by two reviewers (AA and BA) using the Cochrane RoB tool (RoB 2) [8]. This included assessing six bias domains in each RCT: (i) selection, (ii) performance, (iii) detection, (iv) attrition, (v) reporting, and (vi) other bias (including conflict of interest, funding, and baseline imbalance). Disagreements were resolved after consultation with the senior author (DA).

Primary and Secondary Outcomes

The primary outcome of this review was to assess the risk of progression of LGD to HGD or EAC when managed with RFA versus ES. Secondary outcomes were rates of CE-D and CE-IM, adverse events, and predictors of progression with surveillance and response to RFA.

Meta-Analysis of the Included Data

Meta-analysis was performed using RevMan 5.4 [9]. Due to the sparse data, the Mantel-Haenszel statistical method was used to pool the risk ratios of dichotomous outcomes, ensuring a reliable effect size. A random effects model was used to look at differences between studies regarding study populations (like people from different countries, socioeconomic backgrounds, and medical histories), interventions (like length of the procedure and number of RFA sessions), and outcomes (like length of time between follow-up visits). All statistical values are reported with 95% confidence intervals (CIs). Subgroups were meta-analyzed if two or more studies provided data on both groups for an outcome. Outcomes included (a) progression of LGD to HGD or EAC, (b) LGD to HGD, (c) LGD to EAC, (d) post-treatment CE-D, (e) post-treatment CE-IM, (f) durable CE-D, and (g) durable CE-IM. No pooled analysis or meta-regression was performed of predictors of progression/response due to insufficient studies (one per predictor category). If a follow-up RCT is conducted on the same cohort as an initial RCT, data from the follow-up RCT will be selected for meta-analysis using intention-to-treat (ITT) and will replace the initial RCT. This method ensures the inclusion of all participants as assigned initially, regardless of whether they completed the study per protocol. By doing so, we estimate real-world effectiveness, focus on long-term outcomes, avoid data duplication, and minimize selection and attrition bias to ensure more reliable and valid results. Finally, if per-protocol outcomes for both RFA and ES groups are reported in at least two studies, a per-protocol meta-analysis will be conducted. This procedure assesses efficacy under ideal conditions and performs a sensitivity analysis to ensure robustness and validity. Moreover, the overall effect was considered statistically significant if its p-value ≤ 0.05. I² and the p-value were used for evaluating heterogeneity. I² > 50% was regarded as substantial heterogeneity, and a p-value > 0.05 as statistically insignificant heterogeneity, likely due to chance. Finally, publication bias was not assessed via the funnel plot due to the inclusion of fewer than 10 studies.

Results 

Study Selection

**Figure 1:**
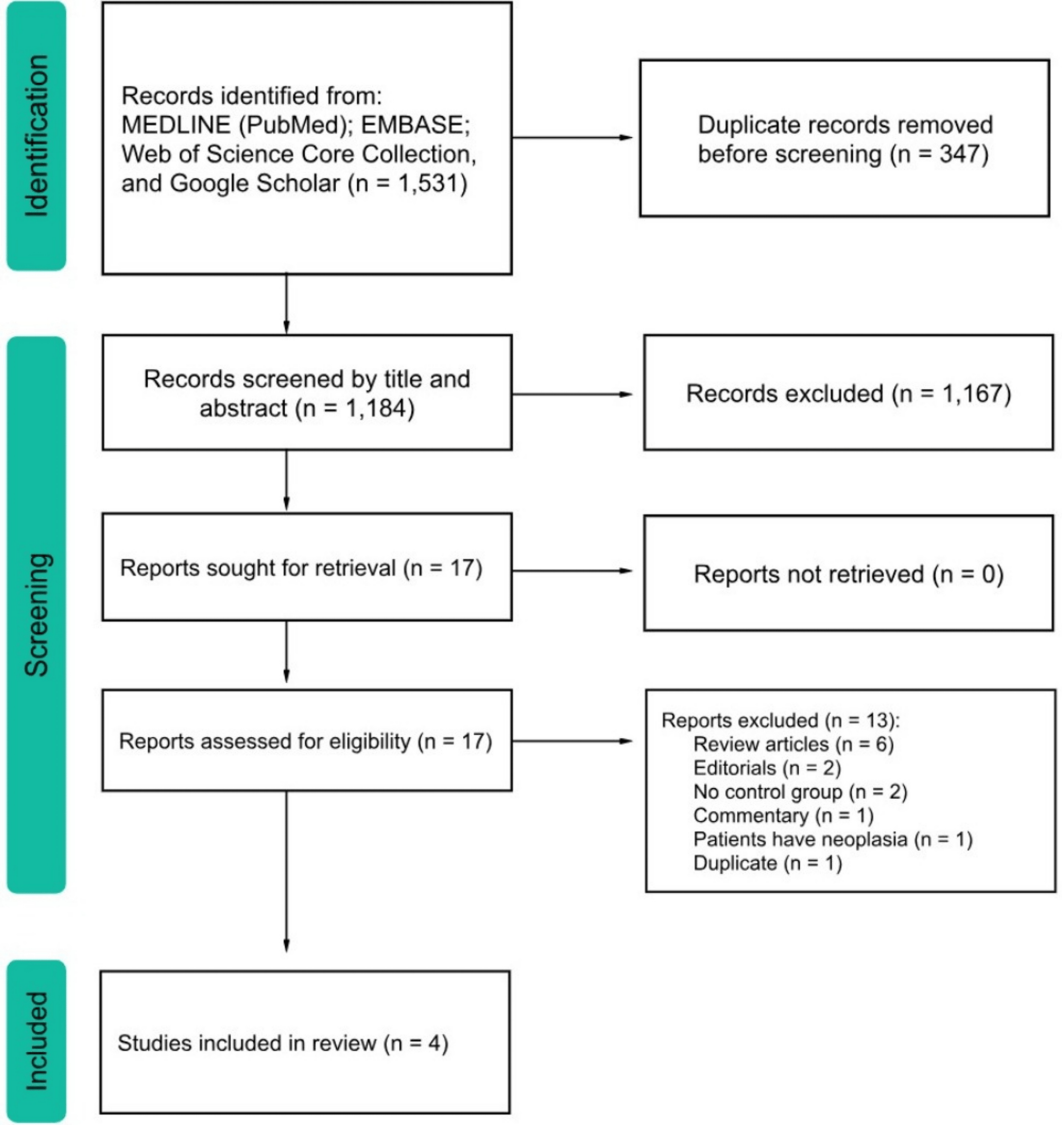
Preferred Reporting Items for Systematic Reviews and Meta-Analyses (PRISMA) flow diagram for the included searches of databases

A total of 1,531 studies were identified from MEDLINE (PubMed) (n = 220), EMBASE (n = 793), Web of Science Core Collection (n = 312), and Google Scholar (n = 206). After removing 347 duplicates, 1,184 studies remained for screening. Following title and abstract screening, 1,167 studies were excluded, leaving 17 papers for full-text assessment. Of these, 13 papers were excluded for not meeting the eligibility criteria, as detailed in the PRISMA flow diagram (Figure 1). Ultimately, four RCT studies were included in this review: three initial RCTs [10-12] and one follow-up RCT [13]. Specifically, Klaver et al. [13] followed up on the patient pool of Phoa et al. [11]. Notably, Shaheen et al. (2011) [14] and Cotton et al. (2017) [15] - follow-ups of Shaheen et al. 2009 [10] - were not included in this review because they did not have an ES (control) group due to crossover.

Characteristics of the Included Studies

Table 1 summarizes the research and patient characteristics of the included studies. Klaver et al. [13] is a follow-up of Phoa et al.'s patient pool [11]. Its median follow-up duration is 48 (interquartile range (IQR) 35-55) months; combined with the former study [11], this results in a total median follow-up of 77 (IQR 64-91) months. All studies excluded patients with nodular BE-LGD. Other common exclusion criteria were age <18 or >80 years, life expectancy less than two years, Barrett’s length >8 cm, prior esophageal therapies, and esophageal comorbidities (e.g., active esophagitis, varices, and strictures).

**Table 1:** Study and patient characteristics of the included studies ^a^ mean ± SD, ^b^ median (interquartile range), ^c^ circumferential length; maximum length Data from Phoa et al.'s (2014) and Klaver et al.'s (2018) studies are presented as initial RCT (Phoa et al.) and long-term follow-up (Klaver et al.). For columns with a single data point, this represents the baseline characteristic from the initial RCT, with no new data reported in the follow-up study. Abbreviations: BMI: body mass index; BE: Barrett’s esophagus; RFA: radiofrequency ablation; ES: endoscopic surveillance; RCT: randomized controlled trial, N/D: no data Shaheen et al. (2009) [10], Phao et al. (2014) [11], Barret et al. (2021) [12], Klaver et al. (2018) [13]

Author, year		Shaheen et al., 2009 [10]	Phoa et al., 2014 [11]; Klaver, 2018 [13]	Barret et al., 2021 [12]
Country		USA	Netherlands	France
Study design		RCT	RCT; long-term follow-up	RCT
Setting		Multicenter	Multicenter	Multicenter
Sample size	RFA	42	68	40
	ES	22	68	42
	Total	64	136	82
Age (year)^a^	RFA	66.3 ± 1.4	63 ± 10	62.8 ± 10.2
	ES	64.6 ± 1.9	63 ± 9	61.8 ± 9.9
Gender (male)	RFA	33/42, 79%	55/68, 81%	36/40, 90%
	ES	19/22, 86%	61/68, 90%	40/42, 95.2%
BMI^a^	RFA	29.2 ± 0.8	26.8 ± 3.7	N/D
	ES	30.9 ± 1.2	27.9 ± 4.8	N/D
Length of BE (cm)	RFA	4.6 ± 0.4^a^	2 (0-6); 4 (2-8)^b,c^	4.0±2.9; 5.6±2.7^a,c^
	ES	4.6 ± 0.5^a^	2 (1-4); 4 (3-6)^b,c^	4.2±3.7; 6.0±3.7^a,c^
Time since diagnosis of BE (years)	RFA	5.8 ± 0.7^a^	5 (2-10)^b^	6.1 ± 5.6^a^
	ES	5.2 ± 1.0^a^	7 (3-11)^b^	5.5 ± 5.0^a^
Time since diagnosis of dysplasia (years)	RFA	2.2 ± 0.5^a^	1 (0-5)^b^	2.2 ± 3.2^a^
	ES	2.4 ± 0.6^a^	2 (0-5)^b^	2.2 ± 2.4^a^
RFA sessions		Up to 4	Up to 5; N/D	Up to 4
Follow-up time (months)		12	36 (30-36); 77 (64-91)^b^	30.0 ± 13.4^a^

Synthesis of Results

**Figure 2:**
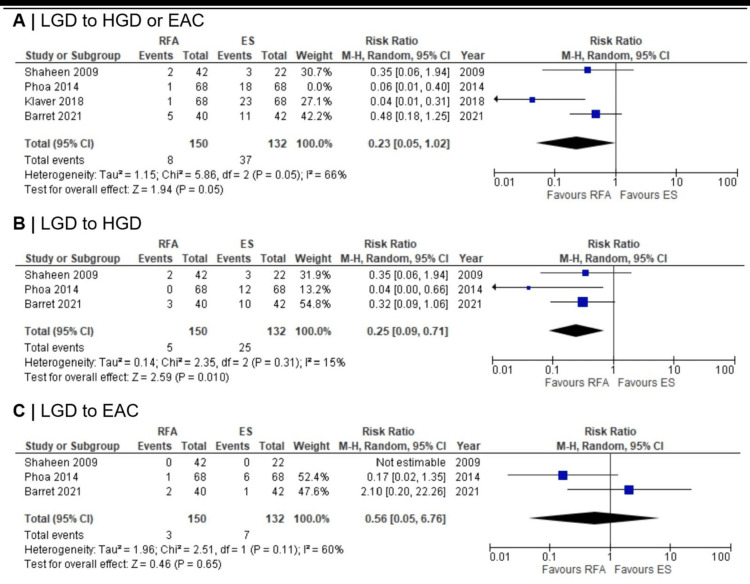
Forest plots comparing RFA versus ES in the progression of LGD using intention-to-treat Follow-up duration in each study: Shaheen et al.: 12 months; Phoa et al.: median (36 (IQR, 30-36) months); Klaver et al.: total median follow-up of 77 (IQR 64-91) months; Barret et al.: 36 months. Note: Klaver et al. (2018) is a follow-up of Phoa et al. (2014). The preserved intention-to-treat analysis of Klaver et al. (2018) covers Phoa et al. (2014) and ensures unbiased results. Abbreviations: IQR: interquartile range, RFA: radiofrequency ablation, ES: endoscopic surveillance, LGD: low-grade dysplasia Shaheen et al. (2009) [10], Phao et al. (2014) [11], Barret et al. (2021) [12], Klaver et al. (2018) [13]

Progression to HGD/EAC:A total of 282 patients with BE-LGD from the four RCTs [10-13] were evaluated for progression to HGD or EAC (Figure 2). Out of 150 patients who received RFA, eight progressed - five to HGD and three to EAC. Among 132 patients under ES, 37 progressed - 25 to HGD and seven to EAC. In Klaver et al.'s study [13], the specific progression to HGD or EAC for five ES patients was not reported. The pooled analysis showed borderline statistical significance for overall progression to HGD or EAC (RR = 0.23; 95% CI 0.05-1.02; p = 0.05). However, the confidence interval’s inclusion of the null value (RR = 1.0) suggests that the true effect could range from a substantial reduction to a marginal increase in risk, indicating substantial uncertainty. The pooled RR for progression from LGD to HGD was 0.25 (95% CI 0.09-0.71; p = 0.01), which was statistically significant, whereas the pooled RR for progression from LGD to EAC was 0.56 (95% CI 0.05-6.76, p = 0.65), which was not statistically significant. The nonsignificant EAC result likely reflects insufficient events (3 RFA vs. 7 ES), resulting in a very wide 95% CI. Only Barret et al. [12] reported per-protocol progression outcomes (4/37 RFA, 11/40 ES), so per-protocol meta-analysis was not feasible.

**Figure 3:**
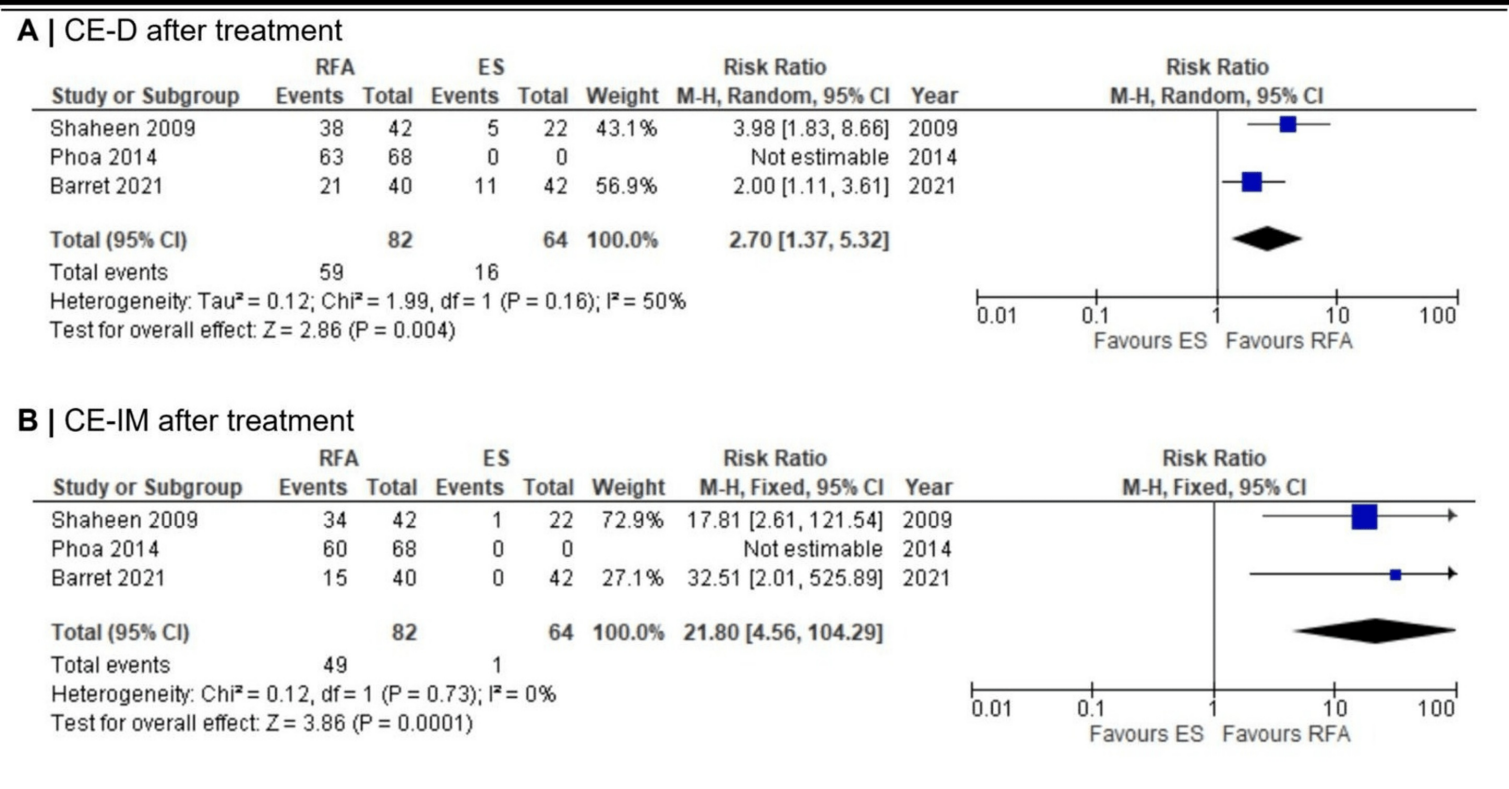
Forest plots comparing CE-D and CE-IM in RFA vs. ES after treatment. Note: Phoa et al. (2014) was not included in the meta-analysis due to the lack of control arm. Abbreviations: CE-D: complete eradication of dysplasia, CE-IM: complete eradication of intestinal metaplasia, RFA: radiofrequency ablation, ES: endoscopic surveillance Shaheen et al. (2009) [10], Phao et al. (2014) [11], Barret et al. (2021) [12]

Treatment response: Three studies [10-12] reported treatment response, defined as CE-D/CE-IM at the first endoscopic assessment three to six months after the final RFA session. However, one study [11] was excluded from the meta-analysis due to missing control arm data. CE-D post-treatment was significantly more likely with RFA compared with ES (RR 2.70; 95% CI 1.37-5.32; p = 0.004) (Figure 3A). Across the two RCTs included in the meta-analysis [10,12], 72% (59/82) of patients in the RFA arms achieved CE-D compared with 25% (16/64) in the surveillance arms. Similarly, CE-IM was markedly higher in the RFA group (RR 21.80; 95% CI 4.56-104.29; p < 0.0001) (Figure 3B).

**Figure 4:**
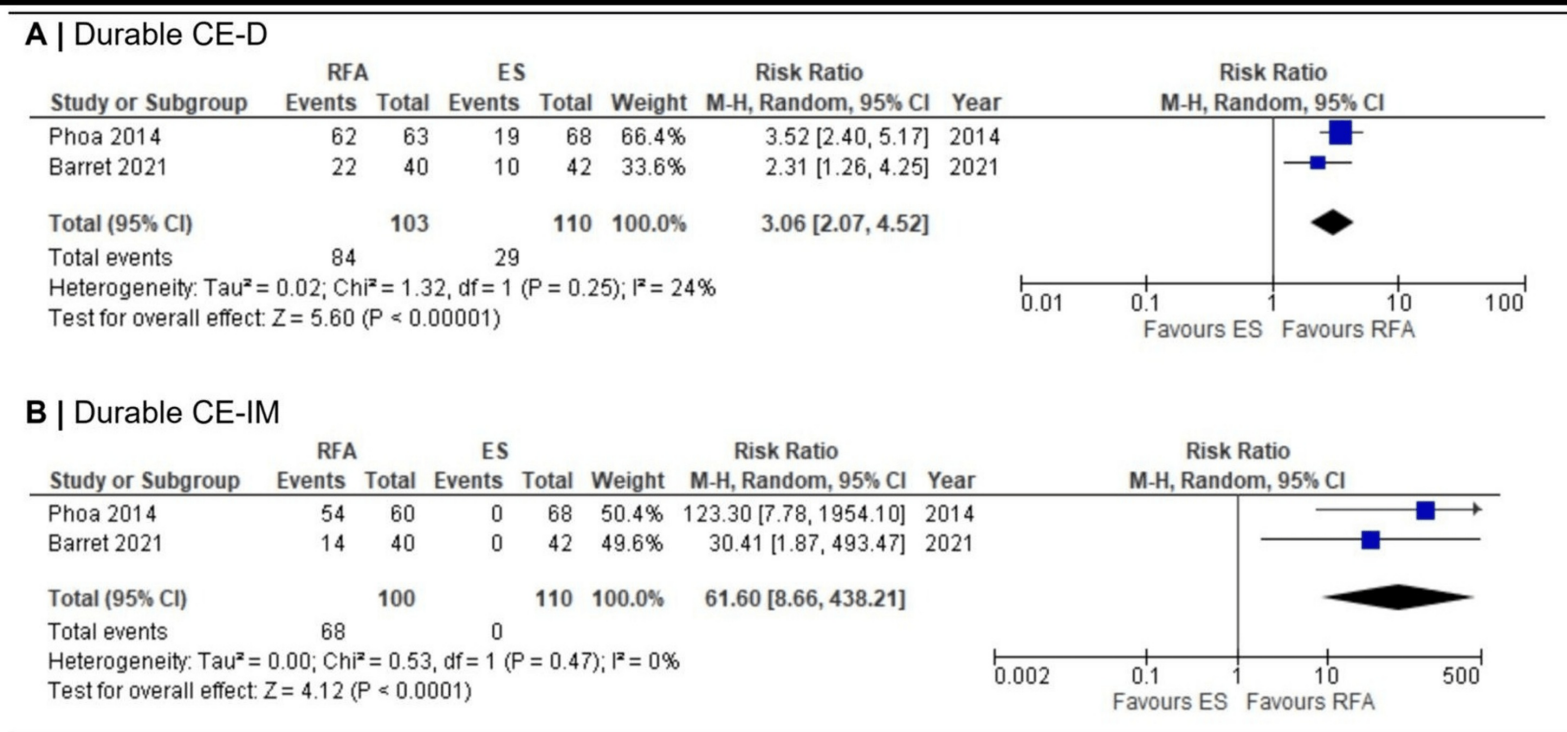
Forest plots comparing durable CE-D and CE-IM in RFA vs. ES at three years Note: The durable CE-IM forest plot scale has been expanded for proper visualization of the wide confidence intervals. Abbreviations: CE-D: complete eradication of dysplasia, CE-IM: complete eradication of intestinal metaplasia, RFA: radiofrequency ablation, ES: endoscopic surveillance Phao et al. (2014) [11], Barret et al. (2021) [12]

Durability outcomes:Durable eradication outcomes from two studies at three years also strongly favored RFA. The pooled risk ratio for durable CE-D was 3.06 (95% CI 2.07-4.52; p < 0.00001) (Figure 4A). Across two RCTs [11-12], 82% (84/103) of patients in the RFA arms maintained CE-D compared with 26% (29/110) in the surveillance arms. Durable CE-IM showed an even greater relative effect (RR 61.60; 95% CI 8.66-438.21; p < 0.0001) (Figure 4B); while the extremely wide confidence interval reflects uncertainty in the precise magnitude of benefit due to no events (0%) in the surveillance group, the entire interval lies far above the null value of 1. This indicates with high certainty that the effect is both large and clinically significant. In these trials, 68% (68/100) of patients treated with RFA maintained CE-IM, whereas no patients (0/110) in the surveillance arms achieved durable CE-IM at three years. Only Barret et al. [12] reported per-protocol durability outcomes at three years (CE-D: 21/37 4/37 RFA, 10/40 ES; CE-IM: 14/37 RFA, 0/40 ES), making per-protocol meta-analysis unfeasible.

Subgroup Analyses

Based on I² and the p-value for heterogeneity, the progression of LGD to HGD or EAC showed borderline significant substantial heterogeneity (I² = 66%; p = 0.05), LGD to HGD showed statistically insignificant low heterogeneity (I² = 15%; p = 0.31), and LGD to EAC showed statistically insignificant moderate-to-substantial heterogeneity (I² = 60%; p = 0.11). For treatment response outcomes, CE-D after treatment demonstrated statistically insignificant moderate heterogeneity (I² = 50%; p = 0.16), while CE-IM after treatment showed no evidence of heterogeneity (I² = 0%; p = 0.73). For durability outcomes, durable CE-D and CE-IM showed statistically insignificant low (I² = 24%; p = 0.25) and absent (I² = 0%; p = 0.47) heterogeneity, respectively. 

Adverse Events 

Three included studies [10-12] reported adverse events, summarized in Table 2. Notably, all studies reported no adverse events in the ES group. Chest pain is the most prevalent adverse event, followed by esophageal strictures (all successfully dilated). Shaheen et al. [10] reported that after initial RFA, the mean chest pain score dropped to 0 by day 8, and after subsequent RFA procedures, it dropped to 0 by day 1. Furthermore, Barret et al. [12] reported that the rate of adverse events decreased gradually with RFA sessions. The studies reported no perforation or procedure-related deaths.

**Table 2:** Adverse events in the RFA group versus ES group ^a^ Median chest-pain score among 40 receiving RFA is 26 (IQR 4–48) on a visual-analogue scale out of 100, with higher numbers reflecting greater pain severity. ^b^ Includes LGD and HGD patients treated with RFA. ^c^ The patient was on antiplatelet therapy. Abbreviations: RFA: radiofrequency ablation, ES: endoscopic surveillance, IQR: interquartile range, LGD: low-grade dysplasia, HGD: high-grade dysplasia Shaheen et al. (2009) [10], Phao et al. (2014) [11], Barret et al. (2021) [12]

Study	RFA		ES
	Mild-moderate adverse events	Severe adverse events	
Shaheen, 2009 [10]	Chest pain on day 1^a ^ 5/84^b ^esophageal stricture	1/84^b ^upper GI bleeding^c^ 1/84^b^ New-onset chest pain eight days after RFA 1/84^b^ Chest discomfort and nausea immediately after RFA	None
Phao, 2014 [11]	1/68 Chest pain three weeks after RFA 8/68Esophageal stricture 3/68 Small mucosal laceration	1/68 Abdominal pain four days after RFA 1/68 Upper GI bleeding seven days after endoscopic resection for a visible lesion (LGD) prior to first ablation. Later, this patient was dilated for stricture and developed a fever and chills.	None
Barret, 2021 [12]	9 (40.9%) Chest pain 1 (4.5%) Esophageal stricture 4 (18.2%) Fever 3 (13.6%) Vomiting 3 (13.6%) Anaesthesia-related 1 (4.5%) Dysphagia 1 (4.5%) Upper GI bleeding	None	None

Single-Study Predictor Findings

While predictors of progression under ES and response to RFA were predefined secondary outcomes, meta-regression or pooled analysis was not feasible because only one study per predictor was identified. These findings are therefore summarized narratively as isolated observations: In Phao et al. [11], multivariable analysis of the ES group showed that a longer duration since BE diagnosis decreased progression risk (OR, 0.84; CI, 0.72-0.98; P-value, 0.02), while more frequent dysplasia-positive endoscopies before baseline and longer circumferential BE length increased progression risk (OR, 1.44; CI, 1.03-2.03; P-value, 0.02 and OR, 1.35; CI, 1.04-1.76; P-value, 0.03, respectively). However, univariate analysis showed age, BMI, maximum BE length, time since diagnosis of dysplasia, Barrett surveillance endoscopies before baseline, and use of proton pump inhibitors had no statistical significance on progression in the ES group. Moreover, in Shaheen et al. [10], initial bivariate analysis suggested that younger age, shorter BE length, lower BMI, and shorter dysplasia history were associated with CE-IM in patients undergoing ablation; however, when subjected to multivariate analysis, these factors were not statistically significant.

Risk of Bias

The RoB assessment of the four included RCTs [10-13] using the Cochrane Collaboration RoB tool [8] revealed one low risk, one high risk, and two unclear risk of bias (Table 3). All four studies had sequence generation, allocation concealment, complete outcome data addressed, and non-selective outcome reporting. Therefore, all four studies scored low in selection bias, attrition bias, and reporting bias. Klaver et al. [13] inherited judgment of selection generation and allocation concealment from Phoa et al. [11], as the same cohort was used with ITT analysis and no mention of re-randomization and post-hoc group changes. Moreover, despite not being explicitly stated, a lack of blinding among patients and personnel is assumed due to cross-overs after randomization, compromising performance bias. The common source of unclear bias was funding and conflict of interest with pharmaceutical and medical technology companies. Finally, as the number of papers was fewer than 10, it was not possible to assess publication bias.

**Table 3:** Authors' judgments on the risk of bias for each included study. Shaheen et al. (2009) [10], Phao et al. (2014) [11], Barret et al. (2021) [12], Klaver et al. (2018) [13]

	Shaheen et al., 2009 [10]	Phao et al., 2014 [11]	Klaver et al., 2018 [13]	Barret et al., 2021 [12]
Sequence generation	Yes	Yes	Yes	Yes
Allocation concealment	Yes	Yes	Yes	Yes
Selection bias	Low	Low	Low	Low
Blinding of patients	Yes	Yes	No	Unclear
Blinding of personnel	Unclear	Yes	No	Unclear
Blinding of outcome assessors	Yes	Yes	Unclear	Unclear
Performance bias	Unclear	Low	High*	Unclear
Detection bias	Low	Low	Unclear	Unclear
Incomplete outcome data addressed	No	No	No	No
Attrition bias	Low	Low	Low	Low
Selective outcome reporting	No	No	No	No
Reporting bias	Low	Low	Low	Low
Other	Conflict of interest and funding	None	None	Conflict of interest and funding
Other bias	Unclear*	Low	Low	Unclear*
Overall risk of bias (RoB)	Unclear*	Low	High*	Unclear *

Discussion

The global prevalence of BE and incidence of esophageal adenocarcinoma are rising, largely driven by persistent risk factors such as obesity and GERD [16]. The AGA guidelines recommend endoscopic resection for BE with visible lesions, via mucosal or submucosal techniques, depending on lesion features. In non-visible BE, management is determined by dysplasia grade; for low-grade dysplasia, a conditional, patient-centered approach is advised, weighing the trade-off between ES and RFA given the limited evidence and lack of consensus [4]. Therefore, this review aims to critically assess the efficacy and safety of RFA compared to ES for managing BE-LGD.

Summary of Key Results

Progression to HGD/EAC: Meta-analysis of four RCTs found RFA showed borderline significant reduction in HGD/EAC progression (RR = 0.23, p = 0.05) with substantial uncertainty (95% CI 0.05-1.02). While progression to HGD was significantly reduced (RR = 0.25, 95% CI 0.09-0.71; p = 0.01), the effect on EAC progression was inconclusive (RR = 0.56, p = 0.65) due to few events (three RFA vs. seven ES) and an extremely wide CI crossing the null value (0.05-6.76). 

Eradication outcomes: Patients receiving RFA were 2.70 times more likely to achieve CE-D shortly after treatment and, most importantly, 3.06 times more likely to maintain it for three years. The effect was even more pronounced for CE-IM, with patients 21.80 times more likely to achieve it initially and a remarkable 61.60 times more likely to maintain it long-term, demonstrating a profound and sustained treatment effect. Notably, spontaneous regression of dysplasia occurred under surveillance alone (25% after treatment and 26% after three years), whereas spontaneous regression of metaplasia was virtually nonexistent.

Adverse events:Safety data showed RFA-associated adverse events (primarily chest pain and strictures) were transient and decreased with subsequent sessions, while no ES adverse events were reported.

Predictors of progression and response:Narrative analysis of single-study predictors identified that under ES, longer BE diagnosis duration decreased progression risk while frequent dysplasia-positive endoscopies and longer BE length increased risk. For the RFA response, initial bivariate associations (age, BE length, BMI, dysplasia duration) did not persist in multivariate models.

Interpretation of Results

Efficacy of RFA: RFA is a highly effective intervention, significantly reducing the risk of progression from LGD to HGD/EAC. Its superiority is further confirmed by high rates of both initial and, crucially, durable CE-D/CE-IM at the three-year follow-up. The markedly higher risk ratio for CE-IM compared to CE-D reflects the near-total inability of surveillance alone to eliminate the underlying metaplastic tissue, whereas low-grade dysplasia can regress spontaneously. Spontaneous regression observed in some ES patients indicates that a subset has indolent disease, creating potential overtreatment risk with universal RFA.

Safety considerations: The absence of adverse events in the ES group across all studies highlights the safe risk profile of ES. By contrast, the presence of adverse events in the RFA group, such as transient chest pain and treatable esophageal strictures, demonstrates the manageable risk profile of RFA. Moreover, the decline in adverse events with repeated RFA suggests improved patient tolerance or operator experience. Finally, the absence of severe complications, such as perforations or procedure-related deaths, reinforces RFA as a relatively safe intervention.

Single-study predictor insights: Single-study analyses suggest longer BE duration under ES is protective, while frequent dysplasia-positive endoscopies and longer BE length increase progression risk. The observed protective effect of longer Barrett’s diagnosis duration may reflect a truly indolent disease course or may be an artifact of length-time bias, wherein patients with longer follow-up inherently represent a lower-risk population. No statistically significant predictors were found for RFA response, indicating that unmeasured patient or disease factors may influence treatment success.

Strengths and Weaknesses of the Included Studies

All included trials were multicenter RCTs, reducing selection bias and strengthening validity [17]. Shaheen et al. [10] and Phao et al. [11] reported double and triple blinding, minimizing performance bias. Follow-up loss was minimal, with non-differential dropout in Shaheen et al. [10], limiting attrition bias. Centralized pathology review further enhanced diagnostic accuracy.

Notable weaknesses were observed. Strict eligibility criteria, excluding nodular BE or segments >8 cm, limit generalizability and may introduce selection bias. Limited RFA sessions may underestimate CE-D and CE-IM rates. Follow-up in early RCTs [10-12] was only one to three  years, insufficient for assessing long-term durability and safety. Barrett et al. [12] reported substantial dropout due to exclusion of patients with neoplastic progression, and Klaver et al. allowed cross-over, likely reflecting the lack of blinding and potential performance bias; ITT analysis mitigated attrition bias. Incomplete per-protocol outcome reporting further constrained the evaluation of efficacy and safety. Conflicts of interest were disclosed in Shaheen et al. [10] and Barrett et al. [12], although their impact is unclear.

According to Tan et al. [18], Barret et al. [12] had several methodological limitations that likely contributed to its less favorable outcomes compared with SURF [11] and EURO-II [19]. First, the trial included centers with variable expertise, many of which did not meet the European criteria for expert BE centers, and outcomes were significantly worse in low-volume sites. Second, the protocol limited patients to a maximum of four RFA sessions, which may be insufficient for long-segment BE, whereas other studies allowed up to five sessions with additional top-up therapy. Third, the esophagogastric junction was not always ablated, leaving a high-risk area potentially untreated, unlike the consistent EGJ ablation performed in SURF [11]. Fourth, the post-ablation PPI therapy was suboptimal, as only one month of double-dose PPI was prescribed before returning to standard dosing, in contrast to the prolonged high-dose PPI regimens used in other trials. Finally, the assessment of CE-IM and CE-D rates after only one year of follow-up may have been premature, not allowing sufficient time for all scheduled ablations to be completed.

*Strengths and Weaknesses of the Study* 

Several efforts were made to strengthen this study's methodology. The study followed the PRISMA guideline to ensure transparency [5]. Moreover, a comprehensive and focused search strategy was formulated using PICO and used in selected bibliography databases and multidisciplinary search engines to ensure adequate recall of pertinent literature. Furthermore, only RCTs were included to ensure minimized confounding bias, lessen heterogeneity, and provide high-quality data. Upon meta-analysis, the random-effect model accounted for inter-study variability in the overall effect, and subgroup analysis was performed to improve generalizability and assess consistency. Moreover, ITT and per-protocol analyses were performed if reported for both groups in two or more studies.

However, there are some notable weaknesses. Restricting inclusion to RCTs, while strengthening internal validity, limited the evidence base and reduced generalizability to real-world practice, where broader cohorts and registries provide valuable safety and effectiveness data. The scarce number of RCTs restricted subgroup analyses and necessitated pooling of studies with heterogeneous follow-up durations (12-77 months), which may affect pooled estimates of long-term outcomes. Per-protocol meta-analysis, although prespecified, was not feasible because fewer than two trials reported outcomes for both intervention and control groups; this reflects a limitation of the included studies rather than our methodology. Furthermore, RFA was not contrasted with alternative therapeutic modalities, which limits the comparative scope of this review. Assessment of publication bias was not possible due to the small number of trials, and some subgroup analyses displayed substantial heterogeneity, further reducing certainty in those estimates.

Comparison With Other Studies

Our findings are consistent with prior RCTs and registry data. Shaheen et al.'s follow-up RCT [14] reported that the annual rate of overall disease progression versus progression to EAC was 2.04% versus 0.51% per patient per year, with durable CE-D maintained in nearly all patients at two to three years. Similarly, Cotton et al. [15] reported that in patients with baseline dysplastic disease (LGD and HGD) achieving CE-IM after RFA, BE recurred in about one-third of patients, mostly within the first year, but the risk of progression thereafter was low, particularly for those who remained BE-free at one year. Real-world data from the Cambridge Barrett’s Registry [20] further support these findings. Among 1,932 patients followed for nearly six years, progression to HGD/EAC occurred at 1.63% per patient-year. Baseline LGD was significantly more common among progressors than non-progressors (22.9% vs. 9.1%), highlighting the elevated risk carried by LGD in routine practice. These findings align with our conclusion that RFA reduces progression to HGD/EAC compared with surveillance. 

The durability of ablation was also demonstrated by Small et al. [21] and the UK RFA registry [22], both showing >80% complete remission of dysplasia rates sustained over long-term follow-up, with cancer progression remaining rare (<2%). Cotton et al. [15] also reported that among patients with baseline LGD, the incidence of BE versus dysplasia recurrence was 8.3 per 100 person-years (95% CI, 4.9-14.0) versus 3.3 per 100 person-years (95% CI, 1.5-7.2). The US RFA registry [23] further confirmed high CE-IM rates (85%), although recurrence of IM occurred in ~20%, typically limited and non-dysplastic. Collectively, these studies reinforce our finding that RFA provides durable eradication in most patients, although ongoing surveillance remains necessary.

Finally, predictors of recurrence and progression identified in external cohorts mirror our observations. Small et al. [21] found nodularity and multifocal dysplasia as independent predictors of progression under ES alone, while the US RFA registry [23] highlighted age, longer BE length, non-Caucasian race, and advanced baseline histology as risk factors for recurrence. These results corroborate our findings on predictors of disease progression and underscore the importance of risk stratification when counseling patients.

Practical Applications

Patients with non-nodular BE-LGD can be stratified as high-risk if they have a more recent BE diagnosis, frequent dysplasia-positive endoscopies, and/or longer circumferential BE length. In high-risk patients, the risk of progression and RFA’s efficacious and durable results outweigh its controllable dangers; thus, physicians should consider using RFA as a first-line therapeutic option. Moreover, given the lack of statistically significant predictors of response to RFA, all patients should be closely monitored to ensure long-term treatment success and early detection of recurrence.

Implications for Research

Longer follow-up RCTs that report ITT and per-protocol outcomes annually are needed to enhance insights into the efficacy and safety of RFA in managing BE-LGD. However, given the proven superiority of RFA over ES for primary endpoints and concerns about patient safety, follow-up RCTs should focus on head-to-head comparisons of RFA with other emerging therapies (e.g., cryoablation, hybrid argon plasma coagulation) to identify the most effective and safe approach for managing non-nodular BE-LGD. To address heterogeneity, high-risk patients can be stratified (e.g., those with a more recent BE diagnosis, frequent dysplasia-positive endoscopies, and/or longer circumferential BE length). Finally, due to the lack of statistically significant predictors of response to RFA, future research should focus on identifying predictors of CE-IM in patients treated with RFA. Multivariable analyses incorporating clinical, endoscopic, and molecular data could help identify factors influencing treatment success.

## Conclusions

This systematic review rigorously assessed the efficacy and safety of RFA compared to ES in managing low-grade dysplasia in BE. The findings suggest that RFA mitigates the progression of BE-LGD to BE-HGD while enhancing rates of CE of dysplasia (CE-D) and CE-IM post-treatment and follow-up. However, given the variable progression of LGD and the potential risk of esophageal complications, treatment decisions should be made with careful consideration. Long-term studies are needed to report annual ITT and PP outcomes and identify predictors of RFA response to better define its role in preventing disease progression. Given RFA's proven superiority over ES and patient safety concerns, future studies should compare RFA with emerging therapies like cryoablation and hybrid argon plasma coagulation to determine the most effective and safe treatment for non-nodular BE-LGD.
